# Minor HIV-1 Variants with the K103N Resistance Mutation during Intermittent Efavirenz-Containing Antiretroviral Therapy and Virological Failure

**DOI:** 10.1371/journal.pone.0021655

**Published:** 2011-06-27

**Authors:** Pierre Delobel, Adrien Saliou, Florence Nicot, Martine Dubois, Stéphanie Trancart, Philippe Tangre, Jean-Pierre Aboulker, Anne-Marie Taburet, Jean-Michel Molina, Patrice Massip, Bruno Marchou, Jacques Izopet, ANRS 106-Window Study Team

**Affiliations:** 1 Service des Maladies Infectieuses et Tropicales, Hôpital Purpan, Toulouse, France; 2 INSERM, UMR1043, Toulouse, France; 3 Laboratoire de Virologie, Hôpital Purpan, Toulouse, France; 4 Laboratoire de Pharmacocinétique et Toxicologie, Hôpital Purpan, Toulouse, France; 5 INSERM, SC10, Villejuif, France; 6 Laboratoire de Pharmacologie, Hôpital Bicêtre, Le Kremlin Bicêtre, France; 7 Service des Maladies Infectieuses et Tropicales, Hôpital Saint-Louis, et Université de Paris Diderot - Paris 7, Paris, France; Massachusetts General Hospital, United States of America

## Abstract

The impact of minor drug-resistant variants of the type 1 immunodeficiency virus (HIV-1) on the failure of antiretroviral therapy remains unclear. We have evaluated the importance of detecting minor populations of viruses resistant to non-nucleoside reverse-transcriptase inhibitors (NNRTI) during intermittent antiretroviral therapy, a high-risk context for the emergence of drug-resistant HIV-1. We carried out a longitudinal study on plasma samples taken from 21 patients given efavirenz and enrolled in the intermittent arm of the ANRS 106 trial. Allele-specific real-time PCR was used to detect and quantify minor K103N mutants during off-therapy periods. The concordance with ultra-deep pyrosequencing was assessed for 11 patients. The pharmacokinetics of efavirenz was assayed to determine whether its variability could influence the emergence of K103N mutants. Allele-specific real-time PCR detected K103N mutants in 15 of the 19 analyzable patients at the end of an off-therapy period while direct sequencing detected mutants in only 6 patients. The frequency of K103N mutants was <0.1% in 7 patients by allele-specific real-time PCR without further selection, and >0.1% in 8. It was 0.1%–10% in 6 of these 8 patients. The mutated virus populations of 4 of these 6 patients underwent further selection and treatment failed for 2 of them. The K103N mutant frequency was >10% in the remaining 2, treatment failed for one. The copy numbers of K103N variants quantified by allele-specific real-time PCR and ultra-deep pyrosequencing agreed closely (ρ = 0.89 *P*<0.0001). The half-life of efavirenz was higher (50.5 hours) in the 8 patients in whom K103N emerged (>0.1%) than in the 11 patients in whom it did not (32 hours) (*P* = 0.04). Thus ultrasensitive methods could prove more useful than direct sequencing for predicting treatment failure in some patients. However the presence of minor NNRTI-resistant viruses need not always result in virological escape.

**Trial registration:**

ClinicalTrials.gov NCT00122551

## Introduction

The remarkable benefits of combinations of antiretroviral drugs in controlling human immunodeficiency virus type 1 (HIV-1) infections can be compromised by the development of drug resistance. Non-nucleoside reverse transcriptase inhibitors (NNRTIs), notably efavirenz, are highly effective and are commonly used to treat HIV-1 infections [1]–[3]. However, HIV-1 has a low genetic barrier to the development of resistance towards these compounds as a result of virus mutations at specific positions on the *pol* gene. The lysine (K) to asparagine (N) mutation at codon 103 (K103N) in particular confers great resistance to efavirenz [4], [5].

Direct sequencing is currently used for routine HIV-1 genotyping [6], but it only detects mutated viruses if they account for more than 10 to 20% of the virus population [7]. Increased rates of virological failure to NNRTI-containing regimen have been observed in subjects previously exposed to NNRTIs, despite the fact that no mutations associated with resistance to these compounds was detected by direct sequencing of the HIV-1 *pol* gene at baseline [8], [9]. These observations suggest that pre-existent but undetected minor populations of mutated viruses contributed to the subsequent treatment failure. More sensitive methods for detecting minor mutated viruses have been developed, the main ones are allele-specific real-time PCR and ultra-deep pyrosequencing [7], [10]–[20]. Such ultrasensitive methods have retrospectively detected minor populations of mutated viruses in baseline virus populations in cases of virological escape to NNRTIs [16], [18], [21]–[23]. However, these methods are not currently used in clinical practice, partly because of technical and costs limitations, but also because their clinical benefit remains to be demonstrated in prospective studies. In addition the agreement between allele-specific real-time PCR and ultra-deep pyrosequencing for quantifying minor mutated viruses has not been assessed. Lastly, the clinically relevant threshold above which minor mutated viruses might significantly influence the virological response to antiretroviral therapy remains unclear.

Various strategies of intermittent antiretroviral therapy have been investigated during the past decade as ways of reducing drug-induced toxicity and costs and improving long-term adherence [24]–[27]. However, repeated treatment interruptions have raised questions about the possible emergence of resistant viruses [28]–[30]. A particular concern is the risk of the virus becoming resistant to efavirenz because of the low genetic barrier of HIV-1 to the development of resistance towards this drug and its long half-life that could result in efavirenz monotherapy if the other drugs of the regimen are more rapidly cleared after treatment interruption.

The randomized multicenter open-label ANRS 106 Window trial compared a fixed intermittent strategy of six cycles of alternating 8 weeks off-therapy and 8 weeks on-therapy with continuous therapy over a 96-week period [24]. NNRTI-associated resistance mutations, as assessed by direct sequencing, increased over time in the plasma of patients in the intermittent arm receiving NNRTIs [31]. We have now evaluated the performances of allele-specific real-time PCR and ultra-deep pyrosequencing for detecting the emergence of minor virus populations harboring the K103N mutation in patients in the intermittent arm receiving efavirenz. We also investigated whether the pharmacokinetics variability of efavirenz influenced the emergence of K103N mutants and analyzed the impact of emergent K103N mutants on the subsequent virological response to combined antiretroviral therapy (cART).

## Methods

### Ethics statement

The protocol was approved by the ethics committee of Toulouse University Purpan Hospital and by the Agence Nationale de Recherches sur le SIDA et les Hépatites Virales (ANRS). All patients gave written informed consent. The study is registered at clinicaltrials.gov, no. NCT 00122551.

### Study subjects

The 21 HIV-1 infected patients had prospectively been included in a substudy of the ANRS 106 trial, on the basis of (i) being in the intermittent arm ; (ii) receiving efavirenz ; (iii) providing informed consent for additionnal blood samples required for the efavirenz pharmacokinetics study. They all had a nadir pre-treatment CD4^+^ T-cell count of ≥100 cells/µL, and a CD4^+^ T-cell count of ≥450 cells/µL at screening, and their HIV-1 RNA had been <200 copies/mL for at least the previous 6 months. They underwent a total of 6 cycles starting with 8 weeks off-therapy, followed by 8 weeks on-therapy. Efavirenz was stopped 7 days before the other drugs at the start of each off-therapy period [24]. Virological success under intermittent antiretroviral therapy was defined as a plasma virus load of ≤400 copies/mL at week 96, at the end of the 6^th^ on-therapy period.

### Sample processing and RNA extraction

Virus particles were pelleted from 1 ml of plasma by ultracentrifugation at 23,500 g for 1 h at 4°C before RNA extraction using the QIAamp viral RNA extraction kit (Qiagen). All the RNA extracted was then used in RT-PCR amplifications.

### Direct sequencing

Genotypic resistance tests were performed on plasma at the end of each off-therapy period. A first RT-PCR amplification step was performed with the QIAgen one step RT-PCR kit (Qiangen) and the following primers: forward 5′-ATTTTCCCATTAGTCCTATT-3′ and reverse 5′-ATGTCATTGACAGTCCAGCT-3′. A nested PCR was then performed with the Expand High Fidelity Plus PCR System (Roche Diagnostics) and the following primers: forward 5′- CCAAAAGTTAAACAATGGCCATTGACAGA-3′ and reverse 5′- AGTTCATAACCCATCCAAAG -3′. Bulk PCR products were sequenced in both directions by the dideoxy chain termination method (BigDye Terminator; Applied Biosystems) on an ABI 3130 DNA sequencer as previously described [31].

### Ultra-deep pyrosequencing

**Figure 1:**
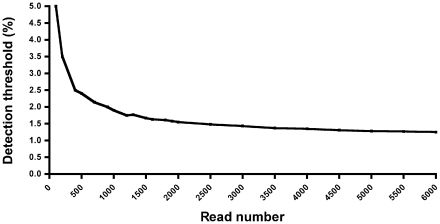
Detection threshold of ultra-deep pyrosequencing for detecting minor K103N variants in function of the read number. The mean error rate of pyrosequencing at codon 103 was 0.0047 [CI99, 0.00085–0.00856]. The upper confidence limit of the error rate was used to calculate the sensitivity of pyrosequencing for a given number of reads. Poisson distribution was used to distinguish authentic variants from artefactual sequences resulting from errors arising during PCR amplification and ultra-deep pyrosequencing. Only those variants whose frequency of occurrence yielded a *P* value of <0.001 according to the Poisson model were considered authentic.

The first RT-PCR amplification step was common for direct sequencing and ultra-deep pyrosequencing. The mean virus load of the samples assesed was 67,792 copies/mL (range 1,200–454,000 copies/mL). A 469-nucleotide long fragment encompassing the reverse-transcriptase gene between codons 88 and 243 was then generated by nested PCR from the RT-PCR products. The nested PCR was performed with the Expand High Fidelity Plus PCR System (Roche Diagnostics). The deep sequencing primers are composed of fusion primers (5′ part) to fuse to the emulsion PCR beads, a four-base “key” sequence to define the DNA library, multiplex identifiers to enable for the identification of samples after pooling and sequencing, and a target-specific sequence (forward 5′-GGAAGTTCAATTAGGAATACCACA-3′ and reverse 5′- TATAGGCTGTACTGTCCATTTGTC-3′). The amplified PCR products were purified using Agencourt Ampure PCR Purification beads (Beckman Coulter) and quantified with the Quant-iT Picogreen dsDNA Assay Kit (Invitrogen) on a LightCycler 480 (Roche). Pooled PCR products were clonally amplified on capture beads in water-in-oil emulsion micro-reactors. A total of 500,000 enriched-DNA beads were deposited in the wells of a full GS Junior Titanium PicoTiterPlate device and pyrosequenced in both forward and reverse directions. The 200 nucleotide flow cycles allow to sequence a 500 bp fragment in a 10-hour sequencing run as about 2.5 nucleotides would be incorporated per nucleotide flow cycle. The sequence reads were quantified with GS Amplicon Variant Analyzer (AVA) software Version 2.5p1 (Roche). The AVA software assigns each read to the proper amplicon and sample using multiplex identifiers. The sequence reads were aligned with the HXB2 consensus sequence. We assessed the frequency of errors resulting from PCR amplification and GS Junior pyrosequencing at codon 103 by analyzing the pyrosequencing data from a panel of 9 plasmid clones previously sequenced by the Sanger method (5 of 9 clones had “AAA” at position 103 encoding “K”, one had “AAG”, also encoding “K”, and 3 had “AAC” encoding “N”). The mean error rate of pyrosequencing at codon 103 was 0.0047 [CI99, 0.00085–0.00856]. The upper confidence limit of the error rate was used to calculate the sensitivity of pyrosequencing for a given number of reads. At least 2500 reads reliably detects minor K103N mutants when they accounted for over 1.5% of the quasispecies. Fig. 1 shows the detection threshold as a function of the read number.

### Allele-specific real-time PCR

**Figure 2:**
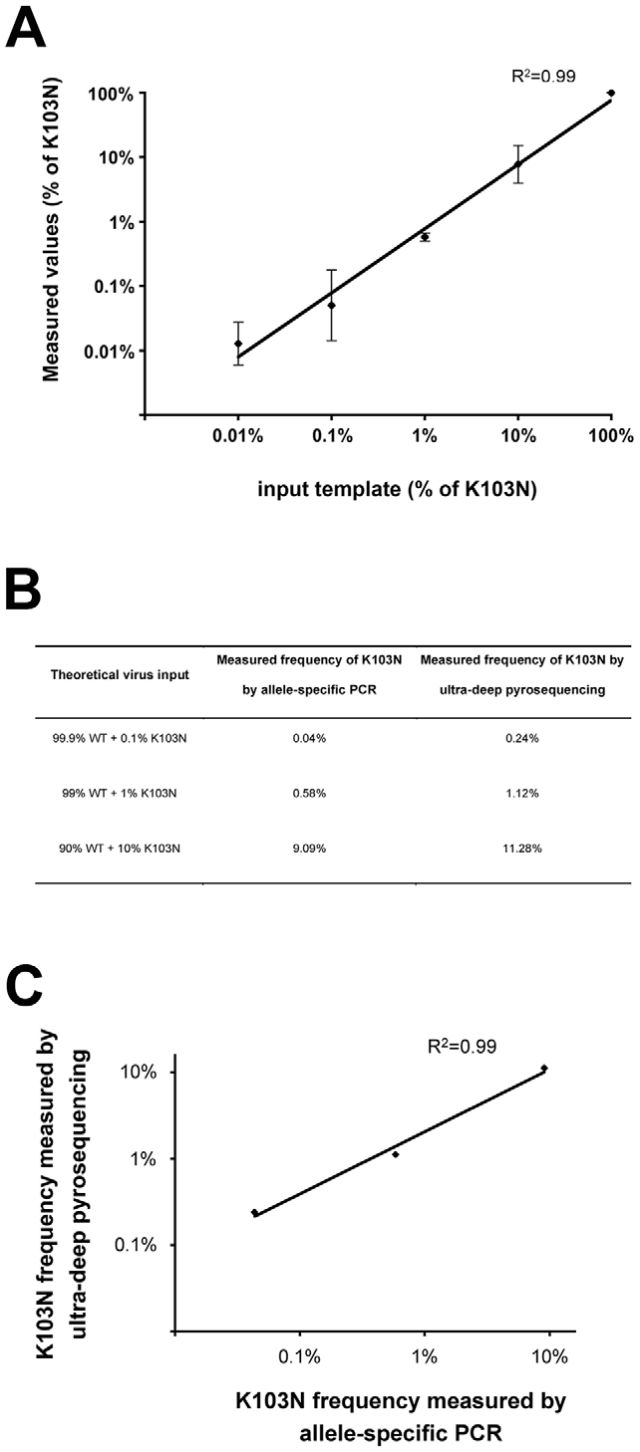
Sensitivity of the allele-specific PCR assay for detecting minor K103N mutants and reciprocal validation with ultra-deep pyrosequencing. **A**. Plot of the measured frequencies of K103N mutants versus the input template, assessed on four independent experiments of mixtures of wild-type and K103N mutants at various frequencies. The inter-assay variability is shown by error bars representing the standard deviation. The assay detects K103N mutants down to a frequency of 0.01% in all experiments. **B**. Measured frequencies of K103N mutants on mixtures of wild-type and K103N mutants with known proportions of K103N. **C**. Correlation between measurements by the two methods.

A first round of amplification was performed with a forward primer (5′-ACATCCCGCAGGGTTAAAAAAGAA-3′) located immediately upstream of the third nucleotide of the 103^th^ codon of the reverse transcriptase gene, and a reverse primer (5′-AGTTCATAACCCATCCAAAG-3′) using the QIAgen one step RT-PCR kit (Qiagen) with the following conditions : 30 min of reverse transcription at 50°C; 15 min of inactivation at 94°C; 30 sec at 94°C, 30 sec at 55°C, and 1 min at 72°C for 40 cycles. The RNA input was adjusted for 50,000 HIV-1 copies per reaction. With this input, the first amplification was linear for 40 cycles (data not shown). This first round of amplification corrected for potential polymorphims of the targeted sequence to ensure that only the third nucleotide of the 103^th^ codon was discriminant in the nested allele-specific amplification. Then 4 allele-specific real-time PCR real-time amplifications were performed in parallel to quantify the AAA and AAG wild-type codons (encoding «K»), and the AAC and AAT mutated codons (encoding «N») using the 4 specific forward primers 5′-CCGCAGGGTTAAAAAAGAIA/G/C/T-3′ and the reverse primer 5′-GGTTCTTTCTGATGTTTTTTGTCTGG-3′ and a Taqman probe 5′-*fam*ATGTGGGTGATGCATATTTTTCAGTTC*tamra*-3′ on a LightCycler (Roche) with the following conditions :10 min at 95°C; 15 sec at 95°C, 1 min at 62°C for 50 cycles. Each quantification was performed using a standard curve obtained by serial dilutions of a plasmid containing one of the targeted AAA, AAG, AAC, and AAT sequences cloned into a pCR4-TOPO vector (Invitrogen). The specificity of each amplification was assessed by amplifying equal quantities of the targeted and non-targeted sequences. The amplification specific for the AAC mutated sequence resulted in 2^20^ and 2^16^ more efficient amplifications of the targeted AAC sequence than the non-targeted AAA and AAG wild-type sequences. The amplification specific for the AAT mutated sequence resulted in 2^29^ and 2^21^ more efficient amplifications of the targeted AAT sequence than the non-targeted AAA and AAG wild-type sequences. The input for quantifying the mutated sequences was adjusted for 5,000 copies per reaction based on the quantification of wild-type sequences. The AAC and AAT amplifications were specific enough to extinguish the wild-type sequences in the amplification. The sensitivity of our allele-specific PCR assay to detect K103N mutants was assessed on mixtures of wild-type and K103N mutants. Our assay detects K103N mutants down to a frequency of 0.01%. This 0.01% threshold of detection was thus used in the study. A plot of the measured values versus the input was linear across a wide range of K103N mutants frequencies (Fig. 2A). Reciprocal validation of allele-specific PCR and ultra-deep pyrosequencing was also performed by quantifying in parallel mixtures of wild-type and K103N mutants with known proportions of K103N by the two methods (Fig. 2B and 2C).

### Quantitative determination of efavirenz in the plasma

We used high-performance liquid chromatography (HPLC) with UV detection to determine efavirenz concentrations in the plasma 1, 3, 7, and 10 days after interrupting efavirenz therapy to assess interindividual variations. Half-lives were calculated from the slope of concentrations decline versus time.

### Statistical analysis

Quantitative variables were compared using the Wilcoxon rank sum test. Correlations between quantitative variables were estimated by calculating Spearman's rank correlation coefficient. Poisson distribution was used to distinguish authentic variants from artefactual sequences resulting from errors arising during PCR amplification and ultra-deep pyrosequencing. Only those variants whose frequency of occurrence yielded a *P* value of <0.001 according to the Poisson model were considered authentic. Statistical analyses were performed with Stata SE 9.2.

## Results

### Patient characteristics at baseline

The 21 patients (15 men, 6 women) had a median age of 38.6 years-old (range, 23.8–59.9), a median CD4^+^ T-lymphocyte count of 648 cells/µL (range, 435–1151), and all had plasma virus load <200 copies/mL. They were all receiving a backbone of nucleoside reverse transcriptase inhibitors associated with efavirenz, plus a protease inhibitor for 3 of them.

### Detection of K103N mutations by allele-specific real-time PCR

The allele-specific real-time PCR to detect K103N mutations could not be performed in 2 patients (harboring wild-type viruses by direct sequencing) because of amplification failure. K103N mutants were detected at the end of an off-therapy period in 15 of the 19 analyzable patients by allele-specific real-time PCR, but in only 6 of these 19 patients by direct sequencing. The frequency of K103N mutants in the quasispecies remained below 0.1% throughout follow-up in 7 of these 15 patients, without further selection. The initial frequency of K103N mutants was 0.1–1% in 2 of the 15 patients, and further increased to become predominant in one of them. The initial frequency of K103N mutants was 1–10% in 4 of the 15 patients, with further selection in 3 of them. Lastly, the initial frequency of K103N mutants was above 10% in 2 of the 15 patients (Table 1).

**Table 1:** Longitudinal assessment of K103N mutants frequency by allele-specific PCR during the intermittent off-therapy periods.

	Frequency of K103N mutants by allele-specific PCR (%)						
Patient	w8	w24	w40	w56	w72	w88	Treatment failure
**1**	0	0	0	0	-	-	**N**
**2**	0	0	0	-	0	-	**N**
**3**	0	0	-	0	-	-	**N**
**4**	-	0	0	0	0	-	**N**
**5**	0.01	0	0	0.01	0	0.03	**N**
**6**	0.03	0.01	0	0.03	-	-	**N**
**7**	0.07	0.05	0.01	-	-	-	**N**
**8**	0	0	0.02	0.01	0.03	0.01	**N**
**9**	0.01	0.01	0	0.02	-	-	**N**
**10**	0	0.01	-	-	-	-	**N**
**11**	0	0.01	-	-	-	-	**N**
**12**	0	0.1	0	0	0	1	**N**
**13**	0	0	0.24	84.7*	72.3*	-	**N**
**14**	0	0	0	0	2.8	-	**N**
**15**	0	0	1.9	29.9*	61.6*	91.9*	**Y**
**16**	0	0	5.8	11*	-	48.4*	**N**
**17**	5*	85.5*	48.7*	66.9*	-	-	**Y**
**18**	-	0	0	0	24.9*	8.9*	**N**
**19**	99.6*	99.2*	-	-	-	-	**Y**

w, week; Y, yes; N, no; *, K103N mutants also detected by direct sequencing; dashes, samples not available.

Allele-specific real-time PCR detected minor K103N mutants in 9 more patients than did direct sequencing; the frequency of K103N mutants was <0.1% in 7 of them, and 1% and 2.8% in the 2 others. The K103N mutants detected by allele-specific real-time PCR in 6 patients were also detected by direct sequencing (Table 1). Allele-specific real-time PCR detected the mutants earlier (week 40) during the selection of drug-resistant viruses than did direct sequencing (week 56) in 3 of them (patients 13, 15, and 16). The frequencies of K103N mutants were 0.24%, 1.9%, and 5.8% in these 3 patients when they were first detected by allele-specific real-time PCR at week 40, and they increased to 84.7%, 29.9%, and 11% when detected later by direct sequencing. Allele-specific real-time PCR and direct sequencing detected K103N mutants at the same time in the 3 other patients (patients 17, 18, and 19). The frequencies of K103N mutants in these latter 3 patients were 5% (at week 8), 99.6% (at week 8), and 24.9% (at week 72) when they were detected by both methods.

The number of K103N copies per mL of plasma was calculated by multiplying the proportion of K103N mutants by the plasma virus load. The absolute numbers of drug-resistant copies in patients with low frequencies of K103N mutants who experienced subsequent selection of their mutated virus populations were 1.54 log_10_ (0.24%, patient 13), 3.65 log_10_ (1.9%, patient 15), 3.97 log_10_ (5.8%, patient 16), and 3.04 log_10_ (5% ; patient 17) per mL plasma when first detected by allele-specific real-time PCR (Table 2).

**Table 2:** K103N mutants in 11 patients quantified by allele-specific real-time PCR and ultra-deep pyrosequencing.

			K103N quantified by allele-specific PCR		K103N quantified by pyrosequencing	
Patient	Stage	Input template (log_10_ HIV-1 copies/ml)	AAC codon	AAT codon	AAC codon	AAT codon
**1**	w40	4.76	0	-	0	0
**2**	w72	5.45	0	0	0	0
**5**	w72	3.70	0	-	0	0
**8**	w72	5.00	0.03% (1.42)	-	0	0
**12**	w8	3.91	0	-	0	0
	w24	3.60	0.1% (0.60)	-	0	0
	w40	3.59	0	-	0	0
	w56	3.83	0	-	-	-
	w72	4.01	0	-	-	-
	w88	3.81	1% (1.81)	0	0	0
**13**	w8	5.18	0	-	0	0
	w24	4.46	0	-	0	0
	w40	4.16	0.24% (1.54)	-	3.7% (2.73)	0
	w56	4.04	84.7% (3.97)	-	38.7% (3.63)	0
	w72	3.87	72.3% (3.73)	0	33.2% (3.39)	0
**14**	w8	4.97	0	-	0	0
	w24	4.89	0	-	0	0
	w40	4.40	0	-	0	0
	w56	4.73	0	-	0	0
	w72	4.16	2.8% (2.61)	0	4.8% (2.84)	0
**15**	w8	5.65	0	0	-	-
	w40	5.37	1.9% (3.65)	0	2.9% (3.83)	0
	w56	4.94	29.9% (4.42)	0	55.1% (4.69)	0
	w72	5.16	61.6% (4.94)	0.05% (1.85)	89.3% (5.11)	0
	w88	5.23	90.2% (5.19)	1.7% (3.46)	79.4% (5.13)	0
**16**	w8	5.66	0	0	0	0
	w40	5.20	5.8% (3.97)	0	5.7% (3.96)	0
	w56	5.01	11% (4.05)	0	17.9% (4.26)	0
	w88	4.98	47.8% (4.66)	0.6% (2.76)	-	-
**17**	w8	4.34	5% (3.04)	-	19.8% (3.64)	0
	w24	3.08	85.5% (3.01)	-	84.5% (3.01)	0
	w40	3.11	48.7% (2.80)	-	80.6% (3.02)	0
	w56	4.65	66.9% (4.47)	0	75.4% (4.52)	0
**18**	w24	3.71	0	-	0	0
	w40	3.65	0	0	0	0
	w56	3.84	0	0	0	0
	w72	3.68	0	24.9% (3.08)	0	10.2% (2.69)
	w88	3.69	0	8.9% (2.64)	0	43.1% (3.33)

The frequencies of K103N mutants are shown with corresponding absolute numbers of K103N log_10_ copies per mL of plasma in brackets.

w, week; AAC and AAT codons encode the asparagine « N » at position 103; dashes, samples not available.

### Detection of K103N mutations by ultra-deep pyrosequencing

**Figure 3:**
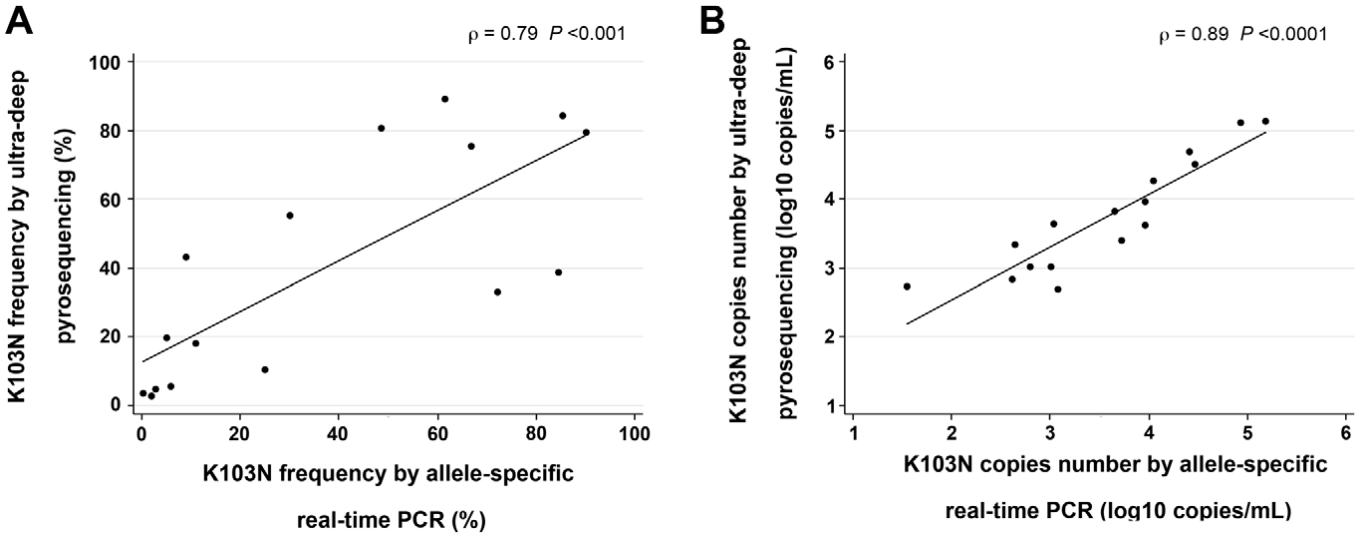
Correlation between K103N mutants quantified by allele-specific real-time PCR and ultra-deep pyrosequencing. A. Frequencies of K103N mutants. B. Number of K103N mutant copies The correlation was estimated by calculating Spearman's rank correlation coefficient for 16 samples successfully quantified by both methods for the AAC and/or AAT mutated codons.

The presence of the K103N mutation was assessed by ultra-deep pyrosequencing in 11 of the patients for whom enough plasma samples were available. An average of 3592 analyzable reads was obtained per sample, which enable minor K103N variants accounting for 1.4% of the quasispecies to be detected (Fig. 1). Ultra-deep pyrosequencing did not detect the K103N mutants that were detected by allele-specific real-time PCR at the same stage in 5 samples (frequencies of 0.03%, 0.05%, 0.1%, 1%, and 1.7%), but higher frequencies were detected by both methods with good agreement (Table 2). The frequencies and copy numbers of K103N mutants quantified in parallel by allele-specific real-time PCR and ultra-deep pyrosequencing were strongly correlated (ρ = 0.79 *P*<0.001, and ρ = 0.89 *P*<0.0001, respectively) (Fig. 3A and 3B).

### Impact of variations in the pharmacokinetics of efavirenz on K103N mutation emergence

**Figure 4:**
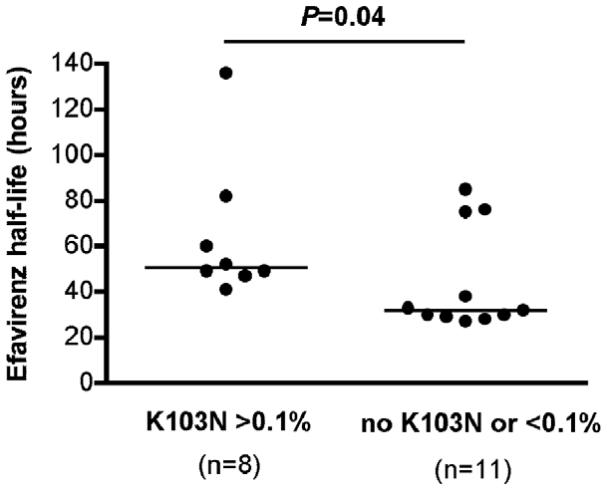
Higher efavirenz half-life in patients in whom K103N emerged than in whom it did not. The Wilcoxon rank sum test was used for the comparison.

Efavirenz was stopped 7 days before the other drugs of the regimen at each off-therapy period to take into account its long half-life. The median concentration of efavirenz after interruption was 1962 ng/ml (range, 728–4146) on day 1, 416 ng/ml (range, 95–1390) on day 3, 112 ng/ml (range, <50–749) at day 7, and 50 ng/ml (range, <50–631) on day 10. Estimations of the half-life of efavirenz revealed great inter-individual variations, from 27 to 136 hours. Thus, even stopping efavirenz 7 days before the other drugs of the regimen could result in efavirenz monotherapy in some patients. The median efavirenz half-life was 32 hours (CI95 [28.7–75.3]) in patients harboring no or few (<0.1%) K103N mutants (n = 11), and 50.5 hours (CI95 [45.1–99.6]) in patients in whom allele-specific real-time PCR detected a significant frequency (>0.1%) of K103N mutants (n = 8) (*P* = 0.04) (Fig. 4).

### Influence of emergent K103N mutations on the subsequent virological response to cART

We analyzed the impact of emergent K103N mutants on the subsequent virological response to cART in the 15 patients in whom K103N mutants had been detected by allele-specific real-time PCR. It had no influence on the virological response to cART in the 7 patients whose frequency of K103N mutants remained <0.1% without further selection. By contrast, the emergence of K103N mutations in the other 8 patients with >0.1% K103N mutants resulted in treatment failure in 3 of them; the remaining 5 maintained a virological response to cART during on-therapy periods (plasma virus load ≤400 copies/mL). K103N mutants were abundant in 2 patients (patients 17 and 19) who were on stavudine, didanosine, and efavirenz, one from the end of the first off-therapy period (99.6%) and the other from the end of the second (85.5%); they experienced treatment failure thereafter. The third patient (patient 15) who experienced treatment failure was on stavudine, lamivudine, and efavirenz. The gradual emergence of K103N mutants was first detected by allele-specific real-time PCR during the third off-therapy period (1.9% at week 40), and the mutated population was then selected at each cycle until the virus population was almost totally mutated (91.9%) at week 88. Treatment failed in this patient after the fifth on-therapy period. By contrast, treatment failure did not occur in 5 patients despite the presence of K103N mutants: the frequency of K103N mutants remained quite low (maximum 1% and 2.8%) in patients 12 and 14, it was high (48.4%) in another (patient 16) who was also given indinavir in addition to efavirenz and lamivudine, and the frequencies of K103N mutants were high (24.9%, and 84.7%) in the remaining 2 patients (patients 13 and 18) who were given only two nucleoside reverse transcriptase inhibitors plus efavirenz. The follow-up data available for these 2 patients showed that they still had an undetectable plasma virus load (<50 copies/mL) 40 weeks after the end of the trial when they were on continuous therapy with the same regimen.

## Discussion

We have assessed the performances of allele-specific real-time PCR and ultra-deep pyrosequencing for detecting minor K103N mutants in patients given efavirenz while on intermittent antiretroviral therapy. An earlier analysis by direct sequencing revealed that the proportion of patients in the intermittent arm given NNRTIs in whose plasma NNRTI-associated resistance mutations were detected increased over time from 3% at the end of the first off-therapy period to 27% at the end of the sixth off-therapy period [31]. We have now retrospectively evaluated two ultrasensitive methods for detecting K103N mutants in this context at high risk for the emergence of resistance.

Allele-specific real-time PCR detected K103N mutants in 9 more patients harboring a low frequency of mutated viruses than did direct sequencing. Allele-specific real-time PCR also provided earlier detection than direct sequencing during the selection of drug-resistant viruses. Plasma samples from 11 patients were assayed by both allele-specific real-time PCR and ultra-deep pyrosequencing. Ultra-deep pyrosequencing is prone to artefactual errors. We have measured the sequence error rate at codon 103 resulting from PCR amplification and ultra-deep pyrosequencing by comparing the GS junior reads to the Sanger sequences of 9 plasmid clones. We used the upper 99% confidence limit of the error rate to calculate the frequency of artefactual sequences for a given number of reads and the Poisson distribution to estimate the number of reads above which K103N mutants found at low frequencies were authentic rather than artefactual. Only those variants whose frequency of occurrence yielded a *P* value of <0.001 according to the Poisson model were considered authentic. Our assay on the GS Junior can detect K103N minor variants that account for 1.5% of total virus if the number of reads is above 2500. But ultra-deep pyrosequencing was not as sensitive as allele-specific real-time PCR for detecting K103N mutants at very low frequency. However, unlike allele-specific real-time PCR, ultra-deep pyrosequencing can determine whether HIV-1 drug-resistance mutations are linked on a given sequence or are borne separately by different virus clones. Globally, the results obtained by allele-specific PCR and ultra-deep pyrosequencing were well correlated, providing reciprocal validation of each method.

We assayed the pharmacokinetics of efavirenz to determine whether its variability could influence the emergence of the K103N mutation. We found that its half-life varied greatly between individuals, from 27 to 136 hours. Thus, stopping efavirenz even 7 days before the other drugs of the regimen could result in efavirenz monotherapy in some patients, a situation in which the risk of NNRTIs resistance emerging is high. This may have occurred as the half-life of efavirenz was significantly higher in the 8 patients in whom K103N emerged than in the 11 patients in whom it did not. However, the small sample size precludes higher levels of statistical significance required for a definite conclusion.

The impact of emergent K103N mutations on the subsequent virological response to cART in the 8 patients harboring >0.1% of the K103N mutated viruses varied. It resulted in treatment failure in 3 patients while 5 paradoxically maintained a virological response to antiretroviral therapy during each on-therapy period. However, 2 of the patients who responded to cART had only a few K103N mutants. A third patient with a high frequency of K103N mutants was given a protease inhibitor in addition to efavirenz, and this could have contributed to the virological response. The last 2 patients had high frequencies of K103N mutants but paradoxically maintained a virological response on efavirenz-based regimen during the intermittent therapy scheme, and on continuous therapy with the same regimen upon completion of the study. Genotypic methods for inferring HIV-1 resistance rely on the presence of known mutations at specific positions of the HIV-1 genome, but cannot assess the impact of changes in the replicative fitness or infectiousness of some viruses [32]. The K103N mutants found in the patients who paradoxically responded to cART might have had altered replicative fitness. This may have occurred as we have previously shown that patients with drug resistant viruses had lower virus rebounds and decreases in CD4^+^ T cells during the period of intermittent therapy than did patients with wild-type viruses [31].

The clinical relevancy of detecting minor populations of mutated viruses remains open to debate. There is growing evidence that the presence of minor mutated viruses at baseline has a deleterious impact on the subsequent response to cART [8], [11], [16], [17], [21]–[23], [33]. However, others found that low frequencies of minor drug-resistant viruses had no impact [34], [35], [36]. Thus, the threshold above which minor mutated viruses might significantly influence the virological response to antiretroviral therapy remains unclear. Spontaneous mutants resulting from random polymorphisms could occur in 0.03%±0.03% of the virus population, in the absence of antiretroviral therapy [37], [38]. Thus, the <0.1% of mutated viruses could be due to spontaneous polymorphisms rather than drug-resistant viruses emerging under drug selective pressure. The possibility of false detection of drug-resistant mutants by ultrasensitive methods cannot also be excluded. The 7 patients harboring <0.1% K103N mutants, as detected by allele-specific real-time PCR, experienced no further selection of their mutated viruses, or treatment failure. By contrast, the mutated virus populations of 4 of the 6 patients harboring 0.1–10% K103N mutants underwent further selection and treatment failed for 2 of these patients. The threshold above which minor K103N mutants could lead to virological escape seemed to be 0.1–1%. A previous report indicated that patients with more than 2,000 (3.3 log_10_) copies/mL plasma of drug-resistant viruses experienced further selection of this population [39]. We found that 3 of the 4 patients in whom drug-resistant viruses subsequently emerged had over 3 log_10_ copies/mL K103N mutants when first detected by allele-specific real-time PCR. The fourth had under 3 log_10_ copies/mL of K103N mutants but nevertheless underwent further selection of the mutated virus population. A recent pooled analysis found that low-frequency NNRTI resistance mutations confer a greater than 2-fold risk of virologic failure in treatment-naive individuals initiating a first-line NNRTI-containing cART regimen. A dose-dependent association of drug-resistant minority variants with increased risk of virologic failure was observed. Absolute numbers of drug-resistant minority variants over 1 log_10_ copies/mL plasma appear to be associated with a statistically significant higher risk of virological failure [40].

In conclusion, we have found that allele-specific real-time PCR and ultra-deep pyrosequencing are more sensitive than direct sequencing for detecting K103N mutants in the particular setting of intermittent antiretroviral therapy, with an excellent correlation between both mehods for quantifying the mutated viruses. These ultrasensitive methods could be more useful than direct sequencing for predicting treatment failure in some patients. But the presence of minor resistant viruses need not always lead to subsequent virological escape. Large prospective studies are now needed to demonstrate the clinical relevance of detecting minor resistant virus populations for adapting antiretroviral therapy.
